# Book Reviews

**Published:** 1914-02

**Authors:** 


### BOOK REVIEWS

CLINICAL DIAGNOSIS AND URANALYSIS.—By James R. Arneill, A. B., M. D., Professor of Medicine and Clinical Medicine in the University of Colorado, and Physician to the Denver County Hospital and the St. Joseph and St. Luke's Hospitals of Denver. New (second) Edition, revised and enlarged. 12mo., 270 pages, with 83 engravings and a colored plate. Cloth, $1.00 net. The Medical Epitome Series. Lea & Febiger, publishers, Philadelphia and New York. 1914.

This work is worth many times its price to the student, the practitioner and the specialist alike. It helps the student to crystalize his impressions, throughout the term as well as at examination time; it enables the practitioner to refresh his memory and keep in touch with all the salient advances, and the specialist to clarify his perspective and readjust his sense of values.

---

DIET IN HEALTH AND DISEASE.—By Julius Friedenwald, M. D., Professor of Gastro-Enterology in the College of Physicians and Surgeons, Baltimore; and John Ruhrah, M. D., Professor of Diseases of Children in the College of Physicians and Surgeons, Baltimore. Fourth Edition, thoroughly revised and enlarged. Octavo of 857 pages. Philadelphia and London. W. B. Saunders Company, 1913. Cloth $4.00. Half Morocco, $5.50 net.

The advancement in this branch of medicine and the changes of opinion have required extensive alterations in this edition. A number of new subjects have been considered. The cream of the literature has been included. The book is practical and clear.

---

PRINCIPLES OF SURGERY.—By W. A. Bryan, A. M., M. D., Professor of Surgery and Clinical Surgery at Vanderbilt University, Nashville, Tenn. Octavo of 677 pages with 224 original illustrations. Philadelphia and London. W. B. Saunders Company, 1913. Cloth, $4.00 net.

Bryan has presented the fundamental facts of surgery in a simple and logical manner. His book should be of great assistance to the student and physician in understanding the principles upon which practical surgery is built. We recommend it most highly and hope in time to come to see more books by our well-known medical and surgical men.

---

A TEXT-BOOK OF THE PRACTICE OF MEDICINE.—By James M. Anders, M. D., Ph.D., LL.D., Professor of Medicine and Clinical Medicine, Medico-Chirurgical College, Philadelphia. Eleventh Edition thoroughly revised. Octavo of 1335 pages, fully illustrated. Philadelphia and London. W. B. Saunders Company, 1913. Cloth, $5.50 net. Half Morocco, $7.00 net.

This work introduces the student to our present knowledge of the practice of medicine in general with special emphasis on the diagnosis, differential diagnosis and treatment. There is no more excellent book in English on the practice of medicine than is Anders'.

---

THE SURGICAL CLINICS OF JOHN B. MURPHY, M. D., at Mercy Hospital, Chicago. Volume II. Number VI. (December). Octavo of 186 pages, illustrated. Philadelphia and London. W. B. Saunders Company, 1913. Published bi-monthly. Price per year, paper, $8.00. Cloth, $12.00.

DORLAND'S AMERICAN POCKET MEDICAL DICTIONARY.—Edited by W. A. Newman Dorland, M. D., Editor "American Illustrated Medical Dictionary." Eighth Edition Revised and Enlarged. 32mo of 677 pages. Philadelphia and London. W. B. Saunders Company, 1913. Flexible leather, gold edges, $1.00 net, thumb index, $y.25 net.

This edition has been carefully revised and a large amount of new matter has been added. The extensive sale of this dictionary has proved its usefulness.

---

A TEXT-BOOK OF PHYSIOLOGY, for Medical Students and Physicians. By William H. Howell, Ph.D., M. D., Professor of Physiology, Johns Hopkins University, Baltimore. Fifth Edition, thoroughly revised. Octavo of 1020 pages, fully illustrated. Philadelphia and London. W. B. Saunders Company, 1913. Cloth $4.00 net. Half Morocco, $5.50 net.

Howell has judiciously limited the material selected and has presented with simplicity and lucidity the essential facts and theories.

---

DIETETICS FOR NURSES.—By Julius Friedenwald, M. D., Professor of Gastro-Enterology in the College of Physicians and Surgeons, Baltimore; and John Ruhrah, M. D., Professsor of Diseases of Children in the College of Physicians and Surgeons, Baltimore. Third Edition enlarged and revised. 12mo volume of 431 pages. Philadelphia and London. W. B. Saunders Company, 1913. Cloth, $1.50 net.

Among the additions to the present edition are the articles on feeding infants, of typhoid fever, scarlet fever, and rectal and duodenal alimentation. This volume will receive the same cordial reception that was given its predecessors.

MATERIA MEDICA, PHARMACOLOGY, THERAPEUTICS AND PRESCRIPTION WRITING.—For students and practitioners. By Walter A. Bastedo, Ph.G., M. D., Associate in Pharmacology and Therapeutics at Columbia University. Octavo of 602 pages, illustrated. Philadelphia and London. W. B. Saunders Company, 1913. Cloth $3.50 net.

This is an adaptation of Bastedo's lectures delivered at Columbia University, and affords an excellent presentation of this subject.

